# Epstein-Barr virus type 2 infection is associated with higher viral loads in pediatric tonsils from western Kenya

**DOI:** 10.1128/spectrum.00740-25

**Published:** 2025-07-08

**Authors:** Emmily Koech, Sidney Ogolla, Asito S. Amolo, Kevin Waomba, Ian Onditi, Katherine R. Sabourin, Bonface Ariera, Stellah Chumbe, Shannon C. Kenney, Rosemary Rochford, Gabriela Samayoa-Reyes

**Affiliations:** 1 Centre for Global Health Research, Kenya Medical Research Institute 118982, Kisumu, Kenya; 2 Department of Biological Sciences, Jaramogi Oginga Odinga University of Science and Technology 259221, Bondo, Kenya; 3 Department of Immunology and Microbiology, School of Medicine, University of Colorado Anschutz Medical Campus 12225, Aurora, Colorado, USA; 4 Department of Oncology and Medicine, McArdle Laboratory, University of Wisconsin School of Medicine and Public Health, Madison, Wisconsin, USA; Oklahoma State University College of Veterinary Medicine, Stillwater, Oklahoma, USA

**Keywords:** Epstein-Barr virus, EBV types, tonsils, Burkitt lymphoma

## Abstract

Epstein-Barr virus (EBV) is an etiologic agent of endemic Burkitt lymphoma (BL), a prevalent pediatric cancer in sub-Saharan Africa. There are two known types of EBV, EBV Type-1 (EBV-1) and EBV Type-2 (EBV-2), both found in eBL patients. To determine the EBV load and type dynamics within the tonsils, saliva, whole blood, and plasma, we enrolled 102 children aged 1–14 undergoing tonsillectomy in a malaria holoendemic region of western Kenya. Additionally, we investigated EBV-2 cell type preference in tonsillar mononuclear cells (TMCs). Saliva, whole blood, and tonsillar tissue were collected at the time of enrollment. Whole blood was centrifuged to separate plasma from red blood cells, and tonsillar mononuclear cells (TMCs) were isolated from tonsils using a ficoll gradient. Using qPCR, we assessed EBV load and type in TMCs, plasma, whole blood, and saliva. Additionally, a subset of samples was subject to cell sorting to determine the infected cell population. We found that EBV loads were significantly higher in TMCs compared to whole blood (*P* < 0.01) and in saliva compared to plasma (*P* < 0.01). Children with EBV-2 exhibited higher EBV loads in TMCs (*P* = 0.016) than those with EBV-1. Both EBV types were detected in T-cells isolated from TMCS and were also observed in different compartments within the same individual. Our findings indicate that EBV-2 infection in tonsils is associated with higher viral loads, consistent with a model where EBV-2 has more lytic replication compared to EBV-1.

**IMPORTANCE:**

This study investigates Epstein-Barr virus (EBV) persistence in children residing in a malaria-endemic region of western Kenya. We examined the dynamic interplay of EBV viral load and type distribution across multiple host compartments: tonsils, saliva, whole blood, and plasma. Our key finding reveals significantly higher viral loads in pediatric tonsils infected with EBV-2 compared to EBV-1, providing novel insights into the phenotypic differences between EBV-1 and EBV-2. This suggests a greater propensity for lytic replication in EBV-2. We also observed the presence of both EBV types within the same individual, but in different compartments. Understanding the dynamics of EBV persistence, particularly the association of EBV-2 with increased tonsillar viral loads, is crucial. This knowledge sheds light on the pathophysiology of EBV infections and may indicate more severe or prolonged infections in affected children, ultimately contributing to improved risk assessment and management of EBV-associated diseases.

## INTRODUCTION

Epstein-Barr virus (EBV) infects the majority of the world’s population where it exists as a life-long persistent infection. However, the virus has been associated with a number of different malignancies including Burkitt’s lymphoma (BL) (1, 2). EBV-associated BL is primarily a pediatric cancer that occurs at high incidence in some countries of sub-Saharan Africa where *Plasmodium falciparum (Pf*) malaria is holoendemic (3). To further our understanding of how EBV emerges to cause malignancy, it is necessary to understand how EBV persists in healthy individuals.

Much of what we know of EBV persistence comes from studies of EBV in healthy adults living in Europe, the United Kingdom, or the United States where there can be a delay of primary infection until young adulthood, all of which show that EBV is shed into saliva throughout the life allowing for onward transmission (4–8). The germinal center theory of EBV persistence was based on healthy US adults and postulates that EBV is transmitted through saliva, transits across the epithelium to infect B-cells in the Waldeyer’s ring of tonsils (5, 6). These EBV-infected B cells emerge out of the tonsil to circulate as latently infected memory B cells in the peripheral blood (5), and viral reactivation occurs when these differentiate to plasma cells. The source of the virus that is shed in the saliva is not well understood, but some argue that the virus released from the plasma cells goes on to infect epithelial cells within the oral cavity (9). A study reported basal lateral to apical transcytosis of EBV across the oral epithelium pointing to a direct link between EBV infection in tonsils and shedding in saliva (10). There are a number of studies that have evaluated EBV in tonsils, but most studies utilize tonsil tissue from individuals living in areas with no malaria transmission (6, 7, 11–14). One study observed higher EBV-infected B cell frequencies in tonsils from a malaria-endemic area of Ghana compared to tonsils isolated from individuals in the United States (15) suggesting that children from malaria-endemic regions have a high EBV load in tonsils. A key unanswered question is does EBV load differ between the different EBV biologic reservoirs (e.g., blood, tonsils, and saliva).

In regions of sub-Saharan Africa with holoendemic malaria, childhood Burkitt lymphoma (BL) incidences are remarkably elevated. This heightened risk is potentially linked to the observation that children frequently become EBV positive before 6 months of age (16) and exhibit high EBV viral loads in their peripheral blood, suggesting compromised viral control. Repeated *Plasmodium falciparum (pf*) infections, prevalent throughout childhood, are hypothesized to contribute to these elevated viral loads. We have proposed a model we term the disequilibrium model of EBV persistence that accounts for the role of *Pf* infections and how repeated *Pf* infections could shift the balance of viral persistence to result in a high viral load and increased risk for BL (17). In this model, the repeated *Pf* infections drive the expansion of EBV-infected B cells through both viral reactivation to plasma cells and reinfection of a new pool of naïve B cells and through proliferation of already infected B cells. In support of this model, we observed that during acute *Pf* infection an expansion of EBV-infected B cells in the systemic circulating blood compartment and an increase in lytic virus in the saliva of children with malaria.

EBV exists as two primary genotypes, EBV Type 1 (EBV-1) and EBV Type 2 (EBV-2), with distinct genetic differences in their EBNA2 and EBNA3C sequence (18, 19). EBV-2 is prevalent in Africa, including Kenya, while EBV-1 is prevalent worldwide (20–22). Although EBV-1 is more efficient at transforming B cells *in vitro*, we have found that EBV-2 exhibits a tropism for T cells (23, 24). In addition, primary B cells transformed with EBV-2 are more lytic than primary B cells infected with EBV-1 as shown both *ex vivo* and in a humanized mouse model (25, 26). Potential mechanisms for the increased lytic reactivation have been focused on the immediate early gene, BZLF1, and its promoter, Zp (25). A single nucleotide variant, called Zp-V3, was shown to result in enhanced lytic activation in EBV (25).

Our study, based in a region of western Kenya in the shores of Lake Victoria, that is known to be holoendemic for malaria and with high BL incidence addresses several questions; does EBV load differ between cell-free (saliva and plasma) and cell-associated (blood and tonsils) compartments and is there a difference in the distribution of EBV genotypes between these compartments? Given the high prevalence of EBV-2, what is the cell type distribution of EBV types in cell-sorted CD3+ T cells and CD19+ B cells isolated from tonsils? We found that tonsils that were infected with EBV-2 had a higher viral load compared to EBV-1-infected tonsils. Interestingly, EBV-1 and EBV-2 were detected in both B and T cells from tonsils, and both EBV types were found in different tissue compartments in the same individual, suggesting a much more dynamic model of EBV persistence than previously appreciated.

## RESULTS

### Study population

This cross-sectional study enrolled a total of 102 children (aged 1–14 years) undergoing tonsillectomy at Jaramogi Oginga Odinga teaching and referral hospital (JOOTRH) in Kisumu, Kenya. Participants were further categorized into two age groups, under 5 years (*n* = 66) and 5 years or older (*n* = 36); these groups were assigned based on BL peak incidence at age 6 and on malaria infection dynamics (27, 28). Young children in highly endemic areas are particularly vulnerable to frequent malaria episodes (4–6 per year), which leads to disease immunity over time (29). While this acquired immunity does not prevent infection, it reduces clinical malaria severity in older children. Detailed demographic and clinical characteristics are presented in Table 1. Figure S1 provides sample collection and analysis details for the study participants.

**TABLE 1:** Clinical and demographic characteristics of study participants overall (*N* = 102)

Characteristic	Value	*P* value
Age in years		
Mean [SD]	4.05 [2.49]	NA^*d*^
Age group (*n*)		
<5 (*n*, %)	66 (64.7)	
≤5 (*n*, %)	36 (35.3)	0.003^*b*^
Average age per group		
<5 (mean [SD])	3.07 [0.09]	
≤5 (mean [SD])	7.14 [2.25]	<0.01^*c*^
Sex (*n*)		
Males (*n*, %)	75 (73.5)	
Females (*n*, %)	27 (26.5)	<0.001^*b*^
Hemoglobin (g/dL)		
Mean [SD]	11.7 [1.28]	
Missing	2 (2%)	NA^*d*^
*Plasmodium* malaria		
qPCR positive (*n*, %)	18 (17.6%)	<0.001^*b*^
qPCR negative (*n*, %)	67 (65.7%)	
Not determined^*a*^ (*n*, %)	11 (17.6%)	
Parasite load (log copies/mL)		
Mean [SD]	4.37 [16.7]	NA^*d*^

^*a*^ No sample available.

^*b*^ Fishers test for comparison of observed proportions between groups.

^*c*^ Unpaired *t* test for comparison of group means.

^*d*^ NA, not applicable.

### Higher EBV viral loads in TMC and saliva compartments

**Fig 1:**
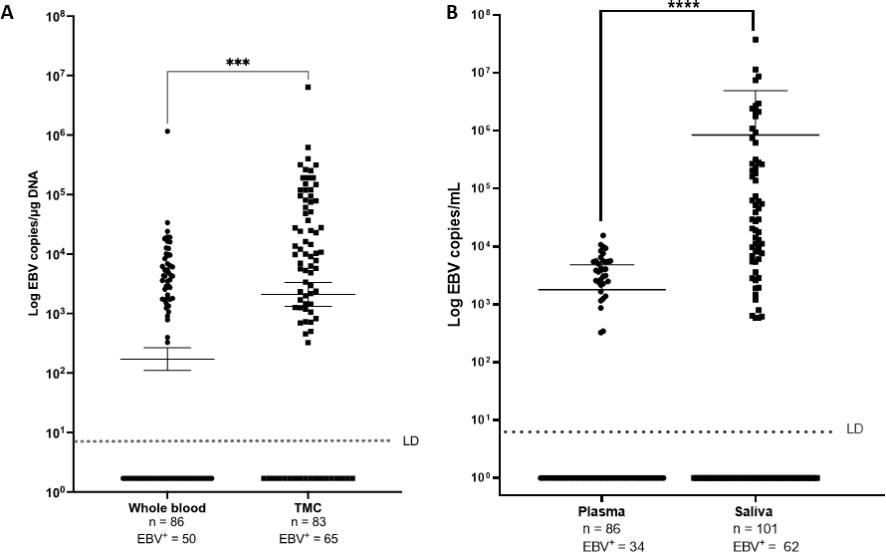
EBV viral load in cell-free and cell-associated compartments. (**A**) Viral load in whole blood and TMC, the cell-associated compartments analyzed ***; *P* < 0.01. (**B**) EBV viral load in cell-free compartments, plasma and saliva ****; *P* < 0.01. Each dot represents an individual sample, and values below the limit of detection are shown.

Cell-associated EBV DNA loads indicative of a latent infection were analyzed in whole blood and TMCs, while saliva and plasma DNA were used to analyze cell-free EBV loads indicative of a lytic infection. EBV viral loads were significantly higher in TMCs compared to whole blood (*P* < 0.01) (Fig. 1A) and in saliva compared to plasma (*P* < 0.01) (Fig. 1B). Percent EBV positive samples were also higher in TMCs (78%) compared to whole blood (58%) and in saliva (61%) compared to plasma (40%).

#### Age-related decreases in EBV viral loads

**Fig 2:**
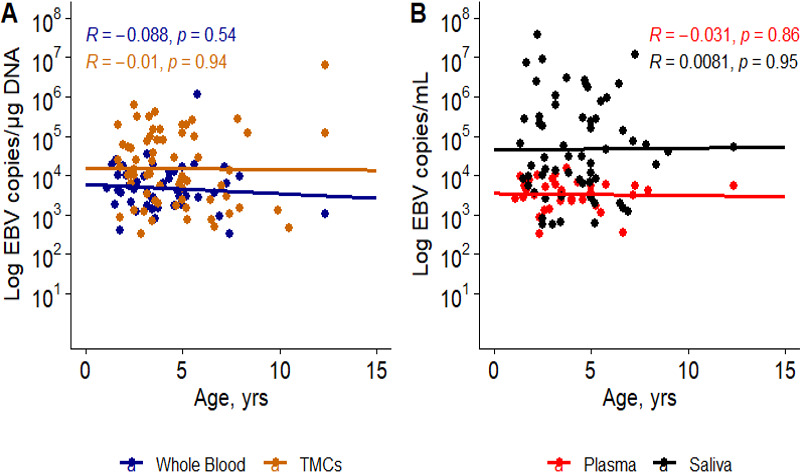
Pearson correlation between EBV viral load and age in cell-free and cell-associated compartments, in children with detectable EBV. (**A**) Correlation of the cell-associated compartments, whole blood (*n* = 50) and TMCs (*n* = 65). (**B**) EBV viral load in cell-free compartments, plasma (*n* = 34) and saliva (*n* = 62).

Because the peak of BL incidence is between 7 and 8 years of age (30), we investigated whether EBV loads differed by age across all four compartments analyzed (TMCs, plasma, saliva, and whole blood). We correlated EBV-positive samples (TMCs *n* = 65, whole blood *n* = 50, plasma *n* = 34, and saliva *n* = 62) with children’s age at time of tonsillectomy. While a trend toward decreasing EBV viral loads with age was observed in whole blood, TMCs, and plasma, this correlation was not found to be significant (Fig. 2A and B).

### Lower EBV viral load in children with asymptomatic malaria

**Fig 3:**
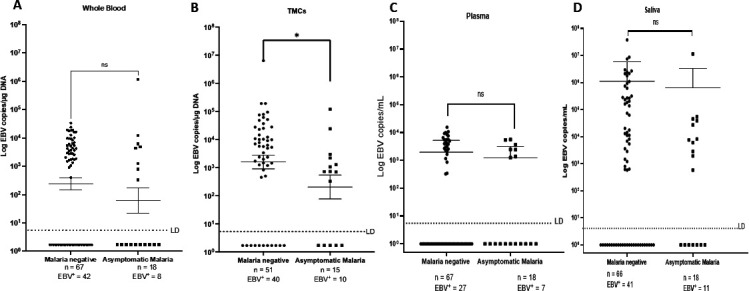
EBV viral load by malaria status; malaria was determined by RDT and BS at time of enrollment, and once sample was collected, qPCR was performed. We found 18 children who had asymptomatic malaria and compared them to those who were *P. falciparum* negative. DNA was isolated from (**A**) whole blood, (**B**) TMCs, (**C**) plasma, and (**D**) saliva and analyzed for EBV viral loads by qPCR.

While all participants had no malaria symptoms and were *Pf* negative by both RDT and blood smear at enrollment, we analyzed whether any of the study participants presented with asymptomatic *Pf* infection by qPCR. We detected that 17.6% of the participants had detectable *Pf* DNA indicating they had an asymptomatic malaria infection. Asymptomatic malaria (*n* = 15) was associated with significantly lower EBV viral loads in TMCs (*P* < 0.05) compared to malaria-negative controls (*n* = 51) (Fig. 3B). Differences in EBV loads in whole blood, plasma, and saliva were not statistically significant (Fig. 3A, C and D).

### EBV-2 is associated with higher EBV viral loads

**Fig 4:**
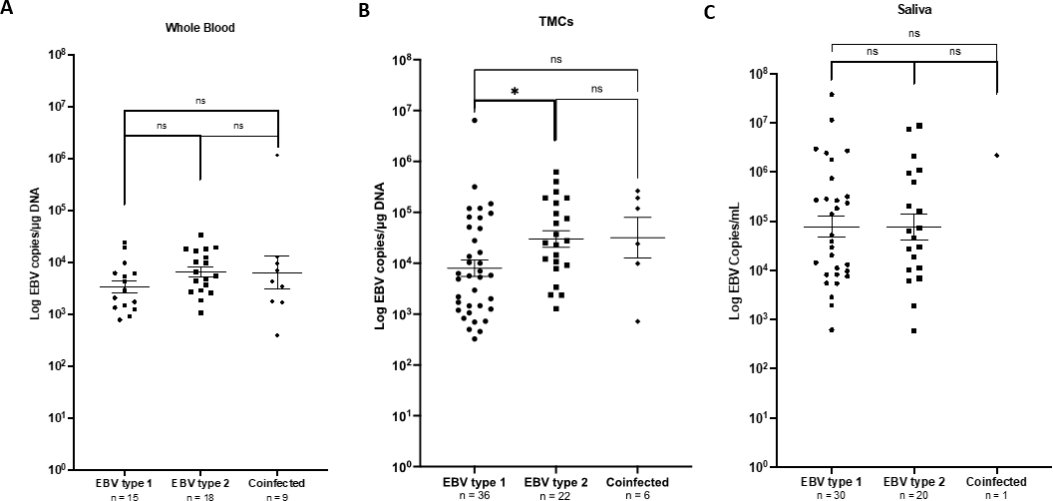
EBV viral load by EBV type. EBV DNA copies were analyzed using qPCR in (**A**) TMCs, (**B**) whole blood, and (**C**) saliva compartments and compared in each EBV type. Samples with undetectable EBV type (whole blood = 8, TMCs = 1, saliva = 11) were not included in the analysis.

To evaluate the EBV type in the EBV+ DNA isolated from blood, TMCs, and saliva, we determined whether samples were infected with either EBV-1 or EBV-2 or were co-infected by evaluating EBNA3C using qPCR. Not all samples had enough DNA concentration to be amplified; these were excluded from further analysis: blood (*n* = 8), TMC (*n* = 1), and saliva (*n* = 11). We observed that in blood, 35.7% of samples were EBV-1, 42.9% of samples were EBV-2, and 21.4% of samples were coinfected. In contrast, in both TMCs and saliva, EBV-1 was the most prevalent with 56.3% of TMC samples containing EBV-1, while only 34.3% were EBV-2 and 9.4% were co-infected. In saliva, 58.9% of samples were EBV-1, while 39.2% were EBV-2 and 1.9% were co-infected. Next, we investigated if EBV viral loads differed based on EBV type. Interestingly, TMCs infected with EBV-2 exhibited significantly higher viral loads compared to those with EBV-1 (*P* < 0.05) (Fig. 4B). A similar trend, although not statistically significant, was observed in whole blood (Fig. 4A).

**Fig 5:**
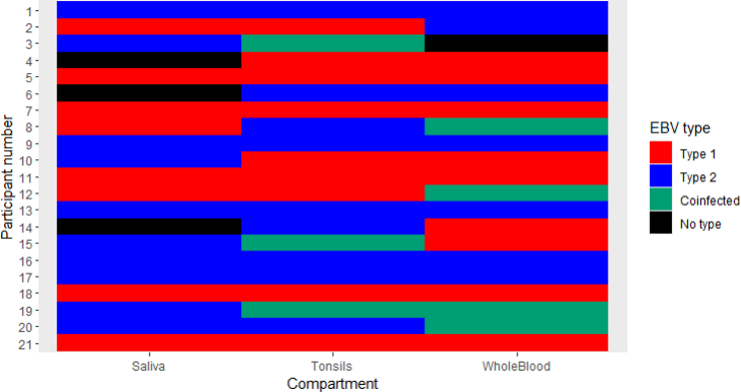
Heat map of EBV type distribution across sample compartments for each individual. This heat map displays EBV type detected in saliva, tonsils, and whole blood from individuals with detectable EBV in all three compartments (*n* = 21). Each color represents a different EBV type: red, Type 1; blue, Type 2; green, co-infection with both types; black, non-typeable. The data demonstrate a roughly equal distribution of EBV Type 1 and Type 2 among participants.

We then determined whether the EBV type was conserved across all analyzed compartments. To do this, we analyzed a subset selected based on the detection of EBV in three compartments in the same participant (*n* = 21). We found that 62% of children had consistent EBV type across all three compartments, while 38% showed discordance of EBV types between compartments (Fig. 5). Of the EBV types detected, 33% were EBV-1, 29% EBV-2, and 38% co-infected. Due to the small sample size, we then analyzed a larger sample number by including children with EBV DNA detected in at least one compartment (*n* = 95). We found that EBV type distribution was inconsistent across compartments. Only 14% of participants had the same EBV type in all compartments analyzed, while most of the participants (86%) had different EBV genotypes across all the compartments (Fig. S2).

### EBV distribution in tonsillar B and T cells

**Fig 6:**
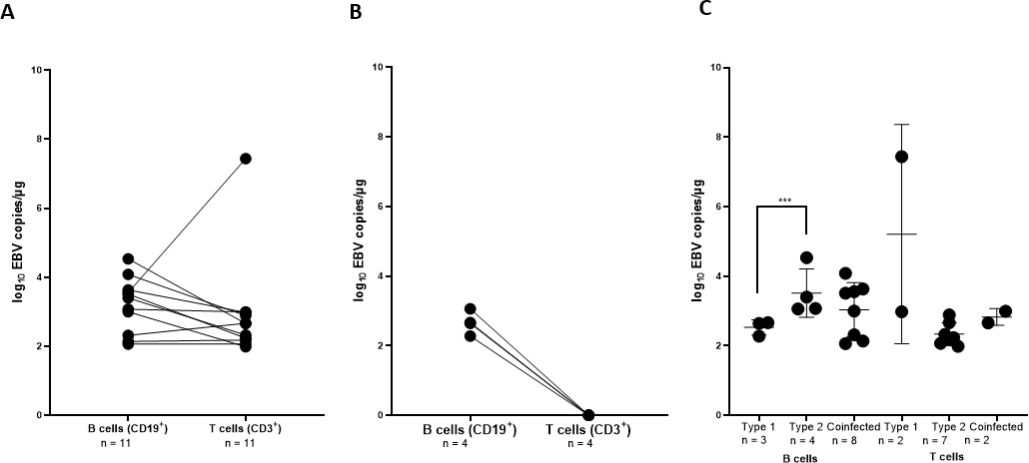
EBV viral load (EBV copies/ug of DNA) is shown in tonsillar B and T cell-enriched fractions. (**A**) Tonsils infected in both B and T cells (*n* = 11). (**B**) Tonsils with infection only in B cells; open squares depict samples with undetectable EBV DNA (*n* = 4). (**C**) All positive TMCs subpopulation were genotyped based on the EBNA3C gene. Here, we present EBV viral load of the different genotypes; EBV type 1, EBV type 2, and coinfection with EBV type 1 and EBV type 2. Each point represents an individual sample; mean and SEM are represented by the bars.

To determine the primary EBV target cell population in tonsils, CD19+ B and CD3+ T cells were sorted from TMCs by fluorescence-activated cell sorting of 15 EBV-positive participants (Fig. S3). Following sorting, the purity of the isolated cells obtained was consistently >99% for each sample. DNA was isolated, and EBV viral load was determined. Results are categorized by samples with detectable EBV DNA in both B and T cells (*n* = 11, Fig. 6A) or only in B cells (*n* = 4, Fig. 6B). Overall, EBV load in B cells was 5.70E + 03 copies/µg of DNA, while in T cells, it was 4.00E + 02 copies/µg of DNA. Notably, 93% of EBV-positive TMCs had higher EBV loads in B cells compared to T cells.

To further investigate the infection of both subpopulations, all EBV-positive samples were typed (EBV-1 and EBV-2) to determine if one genotype preferentially infects a specific cell subtype (Fig. 6C). Interestingly, co-infection with both EBV-1 and EBV-2 was common in B cells (53%), and the highest viral load in B cells was observed in those infected with EBV-2 (*P* < 0.05) (Fig. 6C). EBV-2 was more prevalent in T cells (63%) compared to EBV-1 consistent with a higher tropism of EBV-2 for T cells (23).

## DISCUSSION

Although there are numerous minor variants of EBV described, there are only two major EBV types, EBV-1 and EBV-2 (31). The two types share less than 50% amino acid homology in EBNA-2 and EBNA3s genes (32). These two types are also phenotypically different with greater propensity to lytic reactivation of EBV-2 and greater B-cell transforming ability of EBV-1 (23, 25, 32). But how these genetic and phenotypic differences play in human hosts and how this might alter the risk for EBV-associated malignancies remains unknown. In this study, we analyzed the EBV types in both the systemic (blood, tonsils) and mucosal (saliva) compartments in children living in a region with high risk for BL. We observed that both EBV-1 and EBV-2 were readily detectable in all compartments analyzed; however, EBV-2 had higher viral loads than EBV-1 in TMCs. We found co-infection with both EBV types to be common in this population, and remarkably, different EBV types were often detected in different compartments within the same individual. Furthermore, both EBV-1 and EBV-2 were detected in tonsillar T-cells. These observed dynamics of EBV type distribution across compartments and cell types require us to rethink the model of the EBV life cycle in humans.

We observed that EBV load was higher both in tonsils compared to whole blood and in saliva compared to plasma. Because tonsils are composed of a high percentage of B-cells and are also a known reservoir for EBV infection, we hypothesize this high viral load is due to a higher frequency of infected cells. In addition, ongoing lytic replication could also result in elevated loads. In this regard, we observed higher viral loads in tonsils infected with EBV-2 as compared to EBV-1-infected tonsils. This is consistent with our observations that EBV-2 has a higher propensity for lytic replication either because all EBV-2 types have the Zp-V3 promoter or enhanced activation of the cellular transcription factors, NFATc1 and NFATc2, which increase lytic reactivation in EBV-2 (25, 26). However, a number of other studies using immunohistochemistry to evaluate EBV+ cells and identify EBV transcripts in tonsils have rarely found lytic transcripts (33–35). It is important to note these studies were conducted in regions of the world where there is limited prevalence of EBV-2. Further studies are necessary to evaluate the frequency of lytic replication in tonsil tissues isolated from individuals where EBV-2 is more prevalent.

In our previous studies, we identified that EBV-2 had a T-cell tropism that was not seen with EBV-1. This was observed both following *ex vivo* infection of CD3+ peripheral T cells and in a humanized mouse model (21, 23). We also observed EBV-2 but not EBV-1 in peripheral CD3+ lymphocytes isolated from children in Kenya (36). However, in the current study, we were able to detect both EBV-1 and EBV-2 in CD3+ T cells isolated from tonsils. It is possible that higher levels of CD21 expression on naïve T cells circulating through the tonsils could result in T cells more susceptible to EBV-1 infection. The identification of EBV in tonsillar T-cells is consistent with the studies of Barros et al. (37) who used immunohistochemistry and found EBV EBER+ CD8 and CD4-T cells in tonsils albeit at significantly less frequency than EBV EBER+ B cells.

The identification of both EBV types in different sample compartments of the same individual points to a more dynamic infection. Our results are consistent with the study by Kwok et al. (38), where they evaluated EBV types in 12 patients with infectious mononucleosis and found discordance of viral types between PBMC and saliva. However, they did not have access to tonsils to address differences in that compartment. The results are also consistent with our earlier study following primary infection of Kenyan infants and evaluating EBV types in peripheral blood (22). Co-infection was common with both types, but interestingly, in some study participants, the dominant EBV type changed over time. Together, these data suggest that rather than having a primary EBV infection which results in stable life-long latency in a memory B cell compartment, EBV can flow between different compartments and that different types can reemerge over time.

Repeated *Pf* malaria infections throughout childhood is an etiologic co-factor in the development of BL (39). In this study, none of the participants were symptomatic for malaria nor had parasites in blood as detected by RDT or BS. A subset of the study participants was positive for *Pf* malaria but only detected by very sensitive qPCR assay and would be considered having asymptomatic malaria. Unexpectedly, we observed a decrease in EBV viral load in TMC of asymptomatic *Pf* malaria participants compared to those without any evidence of current *Pf* malaria infection. This inverse association warrants careful consideration. While this finding could represent a novel biological insight, it is crucial to address several factors. These include (i) whether children without evidence of current malaria infection had a recent infection that might have influenced their EBV viral load, and (ii) the immune response in children who are asymptomatic malaria carriers. Specifically, whether acquired immunity in these carriers might elicit only a minimal immune response, insufficient to reactivate EBV, as previously suggested. In the study by Torgbor et al. (15), they found higher EBV loads in tonsils of children from Ghana compared to tonsils from children in the United States, but they did not ascertain current malaria status at the time of sample collection, so it is not possible to compare our results to this study. A limitation of our study is the lack of malaria history on study participants which could impact underlying immune function. Longitudinal studies and comparisons with non-malaria endemic populations are necessary to deepen our understanding of EBV-malaria interactions and their impact on disease outcomes.

Other limitations include the lack of a non-endemic comparison group presents challenges to establish causal inferences in the results from the asymptomatic malaria group. Ideally, we would want to use FACS cell-sorting to identify EBV in B or T cell subsets. However, this presents both a challenge in the number of cells that are feasible to isolate using this technology and the sensitivity of the assay. We were also not able to distinguish between latent or lytic EBV infection. Previously, we used the Epityper methylation assay (17), which can work for saliva but not in DNA isolated where human cellular DNA is present at high levels and overwhelms EBV signal.

In conclusion, our study reveals a dynamic interplay between different compartments of EBV persistence within the human host. The observed elevated EBV-2 loads in tonsils of children contribute to our understanding of EBV-2 and its phenotypic differences from EBV-1. What is paradoxical is why EBV-2 that has a higher propensity for lytic replication is not more prevalent globally. Further investigation into the dynamics of EBV persistence is crucial for understanding and ultimately mitigating the risk of EBV-associated diseases.

## MATERIALS AND METHODS

### Study participants

This study enrolled 102 children (aged 1–14 years), between 2019 and 2022, undergoing tonsillectomy at Jaramogi Oginga Odinga Teaching and Referral Hospital (JOOTRH) in Kisumu County, Kenya, a malaria holoendemic region (40). Inclusion criteria were negative malaria rapid diagnostic test (RDT) (SD-Bioline Korea) and blood smear, hemoglobin >7 g/dL (Hemocue AB Sweden). Exclusion criteria included chronic illnesses such as HIV and diabetes.

### Sample collection and processing

Up to 200 µL of finger-prick blood and saliva were collected prior to tonsillectomy as previously described (41). Blood samples were centrifuged, plasma was separated from whole blood, and volume was replaced using an equal amount of 1× phosphate-buffered saline (PBS). For saliva collection, children were given 5 mL of alcohol-based mouthwash (Johnson & Johnson, NJ, USA) and asked to spit on a 15 mL conical tube. Saliva sample was spun to separate cells from supernatant at 1,500 RPMs for 5 min; the supernatant was used for downstream applications. Blood, saliva, and plasma were stored at −80°C until analysis. Tonsil tissue was obtained during surgery and processed as described earlier (42). Tonsil tissue was processed into a single-cell suspension as previously described (43). Briefly, tonsil samples were weighed, then cut into 3–10 mm fragments, manually homogenized, and transferred into the 70 µm cell strainer to eliminate cell aggregates and connective tissues. Tonsil mononuclear cells were then isolated over density gradient solution using Ficoll Hypaque (Cytiva, USA) and washed two times in complete RPMI 1640 (Sigma-Aldrich, USA). Tonsillar mononuclear cells (TMCs) were cryopreserved in freezing medium (20% FBS, RPMI 1640 [Gibco, USA] and 10% dimethyl sulfoxide [Sigma, USA]). The samples obtained from each study subject are outlined in Fig. S1

### DNA extraction

DNA was extracted from up to 200 µL of whole blood or plasma using DNA mini kit (Qiagen, Germany) as per manufacturer’s instruction with the exception that DNA was eluted in 100 µL buffer AE. A known number of TMC cells was resuspended in 200 µL of 1× PBS (Thermo Fisher Scientific, USA), and DNA was extracted using the Qiagen DNA mini-Kit (Qiagen, Germany). DNA from saliva was extracted using a NucleoSpin kit (Macherey Nagel, Germany), following manufacturers’ instructions. DNA concentration was measured using a Nanodrop 2000 spectrophotometer (Thermo Fisher Scientific, USA).

### *Plasmodium falciparum* DNA amplification using real-time quantitative PCR

*Plasmodial* infection was confirmed using Quantitative real-time PCR (qPCR) targeting the *Pf* 18S ribosomal RNA gene. qPCR reaction details and oligonucleotide sequence have been previously described (44).

### EBV quantification and typing

EBV load was measured using qPCR targeting the BALF5 gene, with β- actin as a reference gene, as previously described (16). EBV type (1 or 2) was determined using EBNA-3C-specific primers and probes (36). Human gamma herpesvirus 4 (HHV-4) type B95-8 (ATCC VR-1492) and Jiyoye [Jijoye, P-2003, P-3J] cell lines (ATCC CCL-87) were used as positive controls for EBV-1 and EBV-2, respectively.

### Tonsillar B and T cell purification by fluorescence activated cell sorting

Cryopreserved TMCs were thawed, washed with complete RPMI 1640 (cRPMI, Sigma-Aldrich, USA), and supplemented with 10% FBS (Gibco, USA). Cells were spun down, and the resulting cell pellet was resuspended in 1× PBS (Gibco, USA). 3–5 × 10^7^ cells were stained for cell sorting, first for viability using Zombie Aqua Fixable Viability dye (Biolegend, USA). Cells were then incubated with Fc blocking reagent (eBioscience, CA) and stained with PE-conjugated anti-CD19 monoclonal antibodies (clone H1B19, Biolegend) and Pacific-blue conjugated anti-CD3 (clone SK7, Biolegend). Cells were resuspended to a final concentration of 5 × 10^5^ cells/mL. The stained cells were sorted using a BD FACSaria cell sorter (BD Biosciences) with an 85 micron nozzle (BD Biosciences), a sheath pressure of 45 pounds per square inch (PSI), and an acquisition rate of 8,000–10,000 events per second. The purity of the sorted fractions was checked after acquisition using 200 sorted cells. DNA was extracted from purified B and T cell populations, and EBV load and type were assessed by qPCR.

### Statistical analysis

Statistical analyses were performed using R (version 4.3.1) and GraphPad Prism (version 8) software. Group means were compared using unpaired *t*-test and one-way analysis of variance (ANOVA), whereas the distribution of proportions was evaluated using Fisher’s exact test. Variables with significant skewness were first transformed toward normality, and statistical associations between EBV viral loads and age were determined using Pearson’s correlation coefficient. *P*-values < 0.05 were considered statistically significant.

## SUPPLEMENTAL MATERIAL

Figures S1, S2, and S3 spectrum.00740-25-s0001.pdf Fig. S1: Flow chart of sample analyzed. Fig. S2: Heat map of EBV type distribution. Fig. S3: Fluorescence-activated cell sorting.

ASM does not own the copyrights to Supplemental Material that may be linked to, or accessed through, an article. The authors have granted ASM a non-exclusive, world-wide license to publish the Supplemental Material files. Please contact the corresponding author directly for reuse.

## References

[1] Womack J, Jimenez M. 2015. Common questions about infectious mononucleosis. Am Fam Physician 91:372–376. 25822555

[2] Wong Y, Meehan MT, Burrows SR, Doolan DL, Miles JJ. 2022. Estimating the global burden of epstein–barr virus-related cancers. J Cancer Res Clin Oncol 148:31–46. doi:10.1007/s00432-021-03824-y 34705104 PMC8752571

[3] Kafuko GW, Burkitt DP. 1970. Burkitt’s lymphoma and malaria. Int J Cancer 6:1–9. doi:10.1002/ijc.2910060102 4319231

[4] Thorley-Lawson DA, Gross A. 2004. Persistence of the epstein-barr virus and the origins of associated lymphomas. N Engl J Med 350:1328–1337. doi:10.1056/NEJMra032015 15044644

[5] Thorley-Lawson DA, Hawkins JB, Tracy SI, Shapiro M. 2013. The pathogenesis of epstein-barr virus persistent infection. Curr Opin Virol 3:227–232. doi:10.1016/j.coviro.2013.04.005 23683686 PMC3789532

[6] Babcock GJ, Decker LL, Volk M, Thorley-Lawson DA. 1998. EBV persistence in memory B cells in vivo. Immunity 9:395–404. doi:10.1016/s1074-7613(00)80622-6 9768759

[7] Chaganti S, Heath EM, Bergler W, Kuo M, Buettner M, Niedobitek G, Rickinson AB, Bell AI. 2009. Epstein-barr virus colonization of tonsillar and peripheral blood B-cell subsets in primary infection and persistence. Blood 113:6372–6381. doi:10.1182/blood-2008-08-175828 19351961

[8] Miyashita EM, Yang B, Babcock GJ, Thorley-Lawson DA. 1997. Identification of the site of epstein-barr virus persistence in vivo as a resting B cell. J Virol 71:4882–4891. doi:10.1128/JVI.71.7.4882-4891.1997 9188550 PMC191718

[9] Hutt-Fletcher LM. 2017. The long and complicated relationship between epstein-barr virus and epithelial cells. J Virol 91:e01677-16. doi:10.1128/JVI.01677-16 27795426 PMC5165189

[10] Tugizov SM, Herrera R, Palefsky JM. 2013. Epstein-barr virus transcytosis through polarized oral epithelial cells. J Virol 87:8179–8194. doi:10.1128/JVI.00443-13 23698302 PMC3700193

[11] Jamiyan T, Nakazato Y, Kuroda H, Kojima M, Imai Y. 2018. Characteristic histological findings of asymptomatic EBV-associated lymphoproliferative disorders in tonsils. J Clin Exp Hematop 58:122–127. doi:10.3960/jslrt.18017 30012922 PMC6408178

[12] Gotoh K, Ito Y, Maruo S, Takada K, Mizuno T, Teranishi M, Nakata S, Nakashima T, Iwata S, Goshima F, Nakamura S, Kimura H. 2011. Replication of epstein-barr virus primary infection in human tonsil tissue explants. PLOS ONE 6:e25490. doi:10.1371/journal.pone.0025490 21998663 PMC3187765

[13] Hug M, Dorner M, Fröhlich FZ, Gysin C, Neuhaus D, Nadal D, Berger C. 2010. Pediatric epstein-barr virus carriers with or without tonsillar enlargement may substantially contribute to spreading of the virus. J Infect Dis 202:1192–1199. doi:10.1086/656335 20815705

[14] Byrne CM, Johnston C, Orem J, Okuku F, Huang M-L, Rahman H, Wald A, Corey L, Schiffer JT, Casper C, Coombs D, Gantt S. 2021. Examining the dynamics of epstein-barr virus shedding in the tonsils and the impact of HIV-1 coinfection on daily saliva viral loads. PLOS Comput Biol 17:e1009072. doi:10.1371/journal.pcbi.1009072 34153032 PMC8248743

[15] Torgbor C, Awuah P, Deitsch K, Kalantari P, Duca KA, Thorley-Lawson DA. 2014. A multifactorial role for P. falciparum malaria in endemic burkitt’s lymphoma pathogenesis. PLOS Pathog 10:e1004170. doi:10.1371/journal.ppat.1004170 24874410 PMC4038605

[16] Piriou E, Asito AS, Sumba PO, Fiore N, Middeldorp JM, Moormann AM, Ploutz-Snyder R, Rochford R. 2012. Early age at time of primary epstein-barr virus infection results in poorly controlled viral infection in infants from Western Kenya: clues to the etiology of endemic burkitt lymphoma. J Infect Dis 205:906–913. doi:10.1093/infdis/jir872 22301635 PMC3282570

[17] Samayoa-Reyes G, Weigel C, Koech E, Waomba K, Jackson C, Onditi IA, Sabourin KR, Kenney S, Baiocchi RA, Oakes CC, Ogolla S, Rochford R. 2024. Effect of malaria infection on epstein-barr virus persistence in Kenyan children. J Infect Dis 229:73–82. doi:10.1093/infdis/jiad264 37433031 PMC10786253

[18] Correia S, Bridges R, Wegner F, Venturini C, Palser A, Middeldorp JM, Cohen JI, Lorenzetti MA, Bassano I, White RE, Kellam P, Breuer J, Farrell PJ. 2018. Sequence variation of epstein-barr virus: viral types, geography, codon usage, and diseases. J Virol 92:e01132-18. doi:10.1128/JVI.01132-18 30111570 PMC6206488

[19] Palser AL, Grayson NE, White RE, Corton C, Correia S, Ba abdullah MM, Watson SJ, Cotten M, Arrand JR, Murray PG, Allday MJ, Rickinson AB, Young LS, Farrell PJ, Kellam P. 2015. Genome diversity of epstein-barr virus from multiple tumor types and normal infection. J Virol 89:5222–5237. doi:10.1128/JVI.03614-14 25787276 PMC4442510

[20] Chang CM, Yu KJ, Mbulaiteye SM, Hildesheim A, Bhatia K. 2009. The extent of genetic diversity of epstein-barr virus and its geographic and disease patterns: a need for reappraisal. Virus Res 143:209–221. doi:10.1016/j.virusres.2009.07.005 19596032 PMC2731007

[21] Coleman CB, Lang J, Sweet LA, Smith NA, Freed BM, Pan Z, Haverkos B, Pelanda R, Rochford R. 2018. Epstein-barr virus type 2 infects T cells and induces B cell lymphomagenesis in humanized mice. J Virol 92:e00813-18. doi:10.1128/JVI.00813-18 30089703 PMC6189503

[22] Smith NA, Baresel PC, Jackson CL, Ogolla S, Toko EN, Heit S, Piriou E, Sumba OP, Middeldorp JM, Colborn KL, Rochford R. 2019. Differences in the epstein-barr virus gp350 IgA antibody response are associated with increased risk for coinfection with a second strain of epstein-barr virus. J Infect Dis 219:955–963. doi:10.1093/infdis/jiy601 30312417 PMC6386812

[23] Coleman CB, Wohlford EM, Smith NA, King CA, Ritchie JA, Baresel PC, Kimura H, Rochford R. 2015. Epstein-barr virus type 2 latently infects T cells, inducing an atypical activation characterized by expression of lymphotactic cytokines. J Virol 89:2301–2312. doi:10.1128/JVI.03001-14 25505080 PMC4338898

[24] Rickinson AB, Young LS, Rowe M. 1987. Influence of the epstein-barr virus nuclear antigen EBNA 2 on the growth phenotype of virus-transformed B cells. J Virol 61:1310–1317. doi:10.1128/JVI.61.5.1310-1317.1987 3033261 PMC254104

[25] Bristol JA, Djavadian R, Albright ER, Coleman CB, Ohashi M, Hayes M, Romero-Masters JC, Barlow EA, Farrell PJ, Rochford R, Kalejta RF, Johannsen EC, Kenney SC. 2018. A cancer-associated epstein-barr virus BZLF1 promoter variant enhances lytic infection. PLoS Pathog 14:e1007179. doi:10.1371/journal.ppat.1007179 30052684 PMC6082571

[26] Romero-Masters JC, Huebner SM, Ohashi M, Bristol JA, Benner BE, Barlow EA, Turk GL, Nelson SE, Baiu DC, Van Sciver N, Ranheim EA, Gumperz J, Sherer NM, Farrell PJ, Johannsen EC, Kenney SC. 2020. B cells infected with type 2 epstein-barr virus (EBV) have increased NFATc1/NFATc2 activity and enhanced lytic gene expression in comparison to type 1 EBV infection. PLoS Pathog 16:e1008365. doi:10.1371/journal.ppat.1008365 32059024 PMC7046292

[27] Kamau A, Mtanje G, Mataza C, Mwambingu G, Mturi N, Mohammed S, Ong’ayo G, Nyutu G, Nyaguara A, Bejon P, Snow RW. 2020. Malaria infection, disease and mortality among children and adults on the coast of Kenya. Malar J 19:210. doi:10.1186/s12936-020-03286-6 32552891 PMC7301992

[28] Hämmerl L, Colombet M, Rochford R, Ogwang DM, Parkin DM. 2019. The burden of burkitt lymphoma in Africa. Infect Agents Cancer 14:17. doi:10.1186/s13027-019-0236-7 PMC6670145 31388351

[29] World Health Organization. 2022. Organisation mondiale de la Santé. 2022. Malaria vaccine: WHO position paper – March 2022 – Rapport mensuel des cas de dracunculose, janvier 2022. Weekly epidemiological record = relevé épidémiologique hebdomadaire. Vol. 97. World Health Organization

[30] López C, Burkhardt B, Chan JKC, Leoncini L, Mbulaiteye SM, Ogwang MD, Orem J, Rochford R, Roschewski M, Siebert R. 2022. Burkitt lymphoma. Nat Rev Dis Primers 8:78. doi:10.1038/s41572-022-00404-3 36522349

[31] Sample J, Young L, Martin B, Chatman T, Kieff E, Rickinson A, Kieff E. 1990. Epstein-barr virus types 1 and 2 differ in their EBNA-3A, EBNA-3B, and EBNA-3C genes. J Virol 64:4084–4092. doi:10.1128/JVI.64.9.4084-4092.1990 2166806 PMC247870

[32] Viel KCMF, Parameswaran S, Donmez OA, Forney CR, Hass MR, Yin C, Jones SH, Prosser HK, Diouf AA, Gittens OE, Edsall LE, Chen X, Rowden H, Dunn KA, Guo R, VonHandorf A, Leong MML, Ernst K, Kaufman KM, Lawson LP, Gewurz B, Zhao B, Kottyan LC, Weirauch MT. 2024. Shared and distinct interactions of type 1 and type 2 epstein-barr nuclear antigen 2 with the human genome. BMC Genomics 25:273. doi:10.1186/s12864-024-10183-8 38475709 PMC10935964

[33] Dias EP, Rocha ML da, Carvalho MO de O, Amorim LM da F de. 2009. Detection of epstein-barr virus in recurrent tonsillitis. Braz J Otorhinolaryngol 75:30–34. doi:10.1016/s1808-8694(15)30828-4 19488557 PMC9442253

[34] Ferressini Gerpe NM, Vistarop AG, Moyano A, De Matteo E, Preciado MV, Chabay PA. 2020. Distinctive EBV infection characteristics in children from a developing country. Int J Infect Dis 93:139–145. doi:10.1016/j.ijid.2020.01.044 32004689

[35] Hudnall SD, Ge Y, Wei L, Yang N-P, Wang H-Q, Chen T. 2005. Distribution and phenotype of epstein-barr virus-infected cells in human pharyngeal tonsils. Mod Pathol 18:519–527. doi:10.1038/modpathol.3800369 15696119

[36] Coleman CB, Daud II, Ogolla SO, Ritchie JA, Smith NA, Sumba PO, Dent AE, Rochford R. 2017. Epstein-barr virus type 2 infects T cells in healthy Kenyan children. J Infect Dis 216:670–677. doi:10.1093/infdis/jix363 28934430 PMC5853903

[37] Barros MHM, Vera-Lozada G, Segges P, Hassan R, Niedobitek G. 2019. Revisiting the tissue microenvironment of infectious mononucleosis: identification of EBV infection in T cells and deep characterization of immune profiles. Front Immunol 10:146. doi:10.3389/fimmu.2019.00146 30842768 PMC6391352

[38] Kwok H, Chan KW, Chan KH, Chiang AKS. 2015. Distribution, persistence and interchange of epstein-barr virus strains among PBMC, plasma and saliva of primary infection subjects. PLoS One 10:e0120710. doi:10.1371/journal.pone.0120710 25807555 PMC4373854

[39] Rochford R, Moormann AM. 2015. Burkitt’s lymphoma. Curr Top Microbiol Immunol 390:267–285. doi:10.1007/978-3-319-22822-8_11 26424650

[40] WHO Global Malaria Programme. 2001. The use of antimalarial drugs: report of a WHO informal consultation, 13–17 November 2000. World Health Organization

[41] Labo N, Marshall V, Miley W, Davis E, McCann B, Stolka KB, Ndom P, Hemingway-Foday JJ, Abassora M, Newton R, Smith JS, Whitby D. 2019. Mutual detection of kaposi’s sarcoma-associated herpesvirus and epstein-barr virus in blood and saliva of cameroonians with and without kaposi’s sarcoma. Int J Cancer 145:2468–2477. doi:10.1002/ijc.32546 31265124

[42] Wohlford EM, Baresel PC, Wilmore JR, Mortelliti AJ, Coleman CB, Rochford R. 2018. Changes in tonsil B cell phenotypes and EBV receptor expression in children under 5‐years‐old. Cytometry Part B Clinical 94:291–301. doi:10.1002/cyto.b.21589 28885784

[43] Johnston A, Sigurdardottir SL, Ryon JJ. 2009. Isolation of mononuclear cells from tonsillar tissue. Curr Protoc Immunol Chapter 7:7. doi:10.1002/0471142735.im0708s86 19653208

[44] Wampfler R, Mwingira F, Javati S, Robinson L, Betuela I, Siba P, Beck H-P, Mueller I, Felger I. 2013. Strategies for detection of Plasmodium species gametocytes. PLoS One 8:e76316. doi:10.1371/journal.pone.0076316 24312682 PMC3848260

