# Supplementary material for: Epstein-Barr virus type 2 infection is associated with higher viral loads in pediatric tonsils from western Kenya
Source: Microbiol Spectr. 2025 Jul 8;13(8):e00740-25. doi: 10.1128/spectrum.00740-25 (PMC12323304; doi:10.1128/spectrum.00740-25)
Supplement: Figures S1, S2, and S3 — Fig. S1: Flow chart of sample analyzed. Fig. S2: Heat map of EBV type distribution. Fig. S3: Fluorescence-activated cell sorting. [file spectrum.00740-25-s0001.pdf]

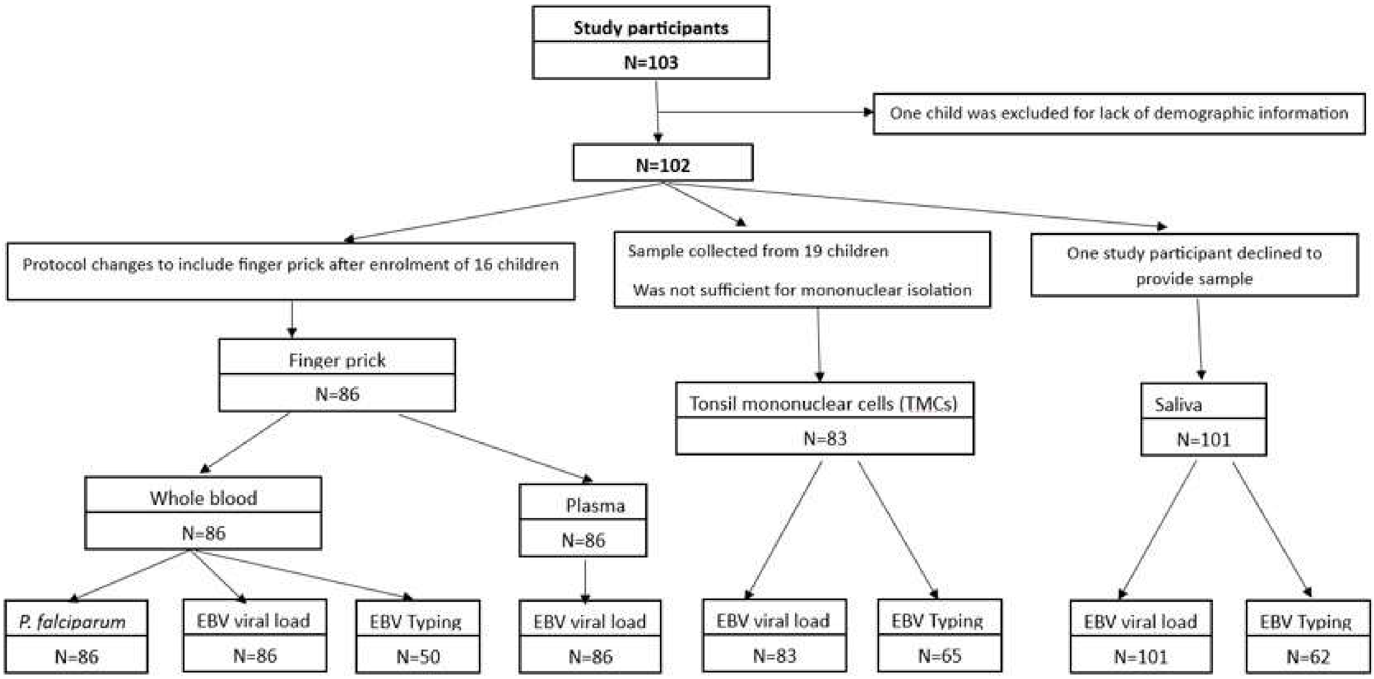

Fig. S1: Flow chart of the samples analyzed; showing sample size of whole blood, plasma, tonsils (TMCs) and saliva compartments.

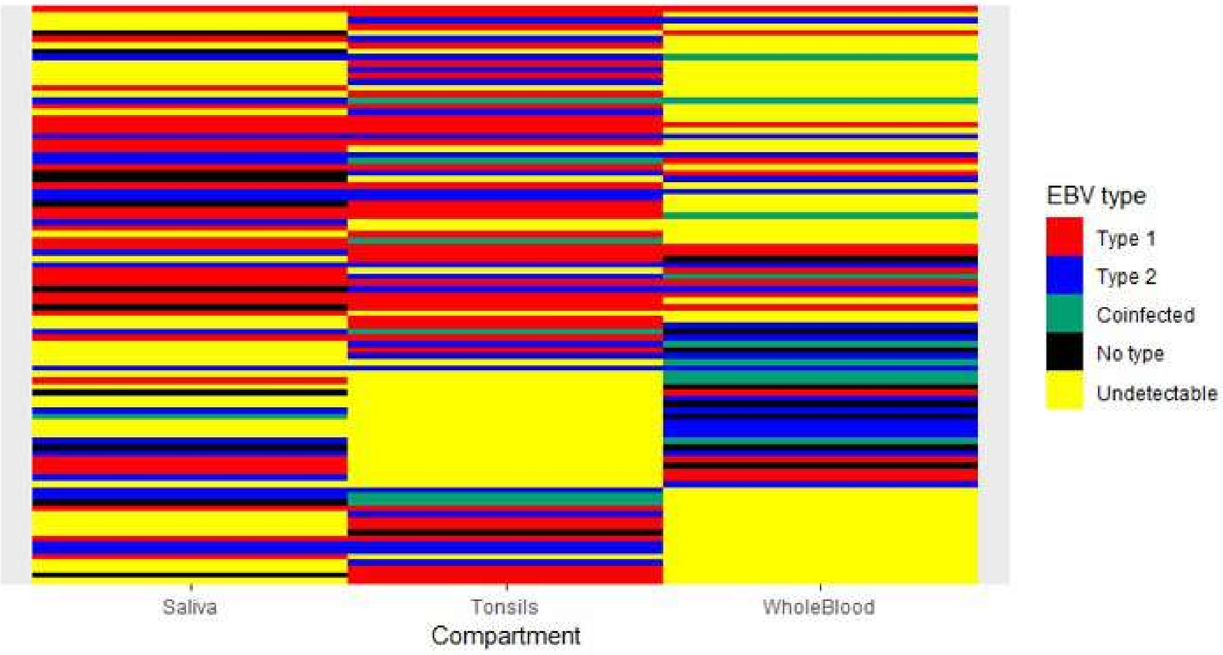

Fig. S2: Heat map of EBV type distribution in 3 sample compartments (saliva, TMCs and whole blood) per individual (n=95). Data shows most of the participants had different EBV genotype across the compartments. Color represents EBV type detected.

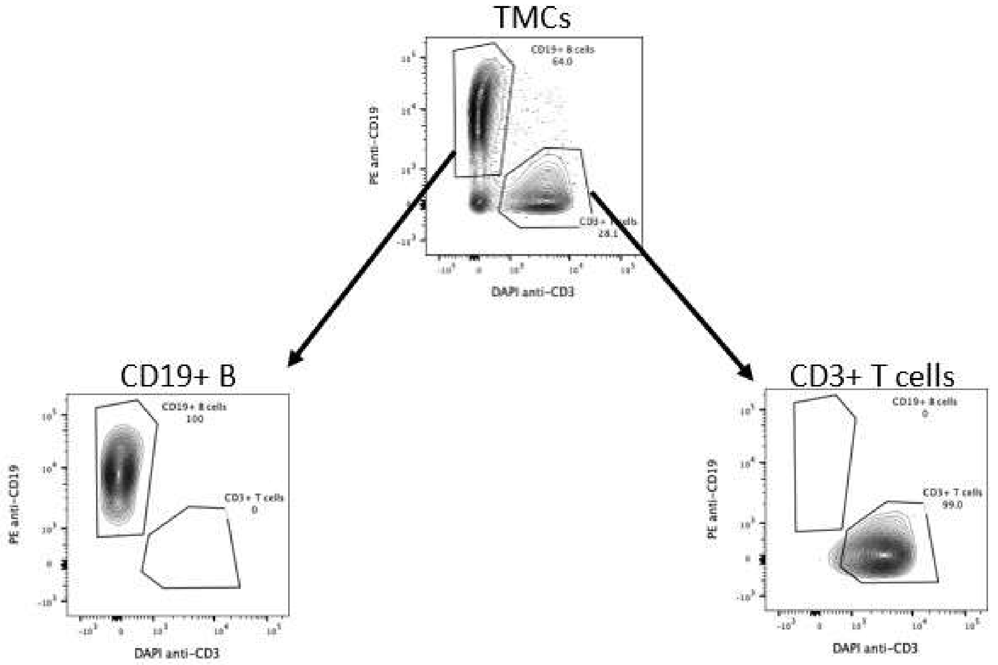

Fig. S3: TMCs from 15 children that were EBV positive were immunosorted by fluorescence-activated cell sorting (FACS). FACS profile and purity (%) of the collected CD3+ T cells and CD19+ B cells are shown.
